# An Exploration of the Maternal Experiences of *Breast* Engorgement and Milk Leakage after Perinatal Loss

**DOI:** 10.5539/gjhs.v8n9p234

**Published:** 2016-01-31

**Authors:** M. Sereshti, F. Nahidi, M. Simbar, M. Bakhtiari, F. Zayeri

**Affiliations:** 1 Departments of Midwifery & Reproductive Health, School of Nursing and Midwifery, International Branch, Shahid Beheshti University of Medical Sciences, Tehran, Iran; 2 Departments of Midwifery & Reproductive Health, School of Nursing and Midwifery, Shahid Beheshti University of Medical Sciences, Tehran, Iran; 3 Departments of Clinical Psychology, School of Medicine, Shahid Beheshti University of Medical Sciences, Tehran, Iran; 4 Department of Biostatistics, School of Allied Medical Sciences, Shahid Beheshti University of Medical Sciences, Tehran, Iran

**Keywords:** perinatal loss, breast engorgement, maternal experiences, milk leakage

## Abstract

**Introduction and Purpose:**

Perinatal loss is one of the toughest events of life. Physiological milk secretion after perinatal loss adds to complicacy of the hardships of the event. The present study is aimed at exploring women’s experience with breast problems and milk leakage after perinatal loss.

**Methods:**

The Study was carried out through explorative quality approach with 18 participants. Sampling method was purposeful and selecting the participants from widest variety was ensured. Data gathering was through deep semi-structured interview and data analyses were done by conventional content analysis. Reliability and validity of the data were ensured by collecting data from a wide range of participants and frequent revisions.

**Findings:**

Data analysis indicated four themes including beyond pain, longing being mother, insufficiency of provided information and coping Strategies, and beliefs and values regarding milk leakage and breast engorgement.

**Conclusion:**

The findings suggested that health care givers needed to inform the patients about probability milk leakage and breast engorgement and remedies to reduce pains and problems of breast engorgement.

## 1. Introduction

Perinatal loss is renowned as one of the worst experiences of life. Not only it means loss of a new baby, but it also signals failure to become a mother, to satisfy maternal affection, and to gain better social and personal status by having a child (Leon, 1992). Furthermore to the loss of a child, these women lose wishes such as feeding their baby, showing love, looking after the infants, and watching his/her growth (Cole, 2012).

The body undergoes considerable physiological and psychological changes during Puerperium to return to pre-pregnancy condition. The period between delivery and the first week afterward is a critical period. Studies have shown that mental and psychological health at this stage has prolonged effects of the mother’s quality of life (Mohseni et al., 2009; Fraiser & Cooper, 2003). Labor pain and the period after that for the women who have had at least one stillbirth experience are almost identical with that of the women without such an experience. However, the former have more risk of encountering negative consequences such as postpartum depression with the effects that might extend over decades (Huberty et al., 2014; Cacciatore & Bushfield, 2007; Blackmore et al., 2011; Vance et al., 2002). A mother who experiences perinatal loss encounters more problems while undergoing natural physiological changes afterward (Cole, 2012). One of these devastating changes is milk leakage and breast engorgement, which is a painful reminder of the loss within few days after delivery.

Lactation is a physiologic and natural process that starts from 16^th^ week of pregnancy and continues after delivery regardless of the birth outcome (Pugmire, 1999; Chen et al., 2015; R. A. Lawrence & R. M. Lawrence, 2014). Milk secretion is a complex mechanism. High level of estrogen, progesterone, and prolactin during pregnancy stimulate anatomic growth of breasts. Prolactin initiates lactose synthesis in breasts while estrogen and progesterone halt it during pregnancy. Dramatic fall of the levels of these hormones after delivery enables prolactin to start milk production (Singh et al., 2009). Prolactin concentration remains high for weeks after delivery even in women who do not breastfeed (R. A. Lawrence & R. M. Lawrence, 2014). Normally, these processes do not result in painful and hard breasts; however, the breast engorgement happens when lactation is more than what is discharged from breasts. Breast engorgement and lack of adequate discharge may create problems such as blockage of milk duct, mastitis, and reduction of milk secretion (Swift, & Janke, 2003). Although, in absence of physical stimulation (e.g. breastfeeding) lactation stops by physiological mechanisms, two-third of women who do not breastfeed develop moderate to severe breast pain and engorgement within 24 to 96 hours after delivery (Spitz, 1998; Humenick & Hill, 1994; Melis et al., 1988; Cheon et al., 2015).

Breast engorgement and milk leakage after perinatal loss are intensified in NICU (neonatal intensive care unit) when such women are encouraged to breastfeed, which stimulate lactation (Bust-Moore & Caitlin, 2003; McGuinness et al., 2014). Physical reminders of perinatal loss such as breast engorgement intensify sense of loneliness and meaninglessness (Callister, 2006; Amato, 2008)

Pain is a physical and psychological stressor, which increases risk of depression as its severity increases. Women experience wide range of pains after delivery, which might be due to delivery problems, breastfeeding problems, or mental trauma. These pains naturally reduce pain coping thresholds (Kendall-Tackett, 2007). Medicine/non-medicine-based interventions or milk donation are usually recommended to cut the side effects. Lactation control medicines are not free of potential side effects (Oladapo & Fawole, 2012), which make them not a best choice for some expert groups. Therefore, non-medicine approaches are more welcomed in some countries and many rely on traditional interventions (Cheon et al., 2015).

Despite several studies on lactation control methods, there is no consensus on a best method and there is no international instruction as to the best approach (Oladapo & Fawole, 2012). Some authors reported that healthcare providers have failed to provide adequate information and appropriate support on suppressing milk production and breast engorgement. In absence of reliable information, women may follow methods that actually backfire (Hillenbrand, & Larsen, 2002). Comprehensive health care services play a key role in initiation of normal and natural grief after perinatal loss (Leoni, 1997). Studies have suggested that mothers who do not receive early consultations about the lactation developed more signs of fatigue and anger. Mastitis may be increased due to high levels of maternal Stress and fatigue after perinatal loss (Fetherston, 1998; Swift & Janke, 2003; Cole, 2012). In addition to physical pain, breast problems create psychological problem that may lead to lack of lactation in future pregnancies. (Cole, 2012; Pugmire, 1999)

When the health care providers have good knowledge of natural physical changes after delivery and psychological problems of perinatal loss, they could be of more help to the mothers (Cole). Taking into account that good and ill health are not mere biological matters and have to do with cultural and social factors; wellbeing is the locus of social values of human society. People and communities approach to health and treatment matters based on their values, norms, and culture (Tavakoli & Naseri Rad, 2010). Necessity of providing support for grieving mothers is undeniable need given emotional and religious concerns and it should be provided by the health care providers who provide health care to mothers after perinatal loss (Busta-Moore & Catlin, 2003). There are few researches on the effects of perinatal loss on maternal lactation. Cultural, social, and economic differences of every society call for separate studies for different societies. No such study has been carried out in Iran thus far. Thereby and in light of absence of a standard guideline and lack of enough knowledge in this field and the mother’s role in management of pain and other breast problems, the present study is aimed at exploring the maternal experiences with the breast engorgement and milk leakage after perinatal loss.

## 2. Material and Methods

The qualitative study was carried out through qualitative content analysis from May 2014 and June 2015. Sampling was carried out purposefully and through snowball method until data saturation (n = 18). The inclusion criteria included: Iranian women with a history of late abortion, stillbirth and neonatal loss experience who suffered from milk leakage and breasts engorgement. The participants had no mental illnesses. Research setting was the health center and hospitals affiliated with Shahid Beheshti and Shahrekord medical science universities. The data was collected through deep semi-structured interview. Before initiating the interviews, the participants were informed that they might leave the study whenever they want and that their information will remain confidential. Maximum possible variability of the participants regarding type of loss, age, number of delivery, prior experiences, education, and job and so on was ensured. The interviews were performed privately with prior arrangement in a clam and decent environment. The interviews were recorded by a voice recorder. The participants were, at first, asked to give demographical information followed by an open-ended question (e.g. let’s talk about the problems after the delivery) and explorative questions afterward. Nonverbal cues such as tone, silence, emphasis, shedding tears and so on were also written down. Each interview lasted between 20 and 60min and immediately afterward, the recoded interview was checked along with the notes and then transcribed. Next interview would be started after analyzing the content of the previous one. Data analyses were carried out short after the interview using conventional content analysis methods.

Data analysis was based on Braun and Clarke’s six steps method. (Braun, & Clarke, 2006)

1. Examining the data: data analysis unit was a whole interview. The transcribed interviews were reviewed for several times and qualitative content analyses were performed.
2. Primary codes: the primary codes were extracted from the interview and then the next interview was carried out. The researchers frequently checked coding process and to ensure validity of the codes and data, the research team and the interview texts were consulted. Afterward, congruity of the coding and the interview was controlled and the codes were compared through reviewing the codes and extracting repeated patterns. Then, the codes were categorized based on similarities and differences. To highlight differences between the categories, continuous comparison was carried out.
3. Extraction of theme: categories with interpretive and epistemological similarities were combined to create a larger category, which was named based on the content.
4. Reviewing and controlling themes based on the data: To ensure authenticity and validity of the themes, traces of data were checked in all main and secondary categories and analyses. The purpose of this process was to minimize number of conceptual units and have more abstract and conceptual main/secondary classifications. Data analysis was continued until further combination or adding abstract concepts was not possible.
5. Defining and naming the themes: the themes were named based on the data under each theme.
6. Producing the report: To check reliability and validity of the data, Lincoln and Guba’s criteria were used (Polit, & Beck, 2003).

Data analyses were performed simultaneously to ensure deep and comprehensive interviews, thorough examination of the information, conditions and methods of the research.

### 2.1 Ethical Considerations

Before starting the interviews, that participants were given insight to the purposes of the study, and confidentiality of the information. In addition, they were given the option to leave the study at whatever stage. Interviews were performed privately with prior arrangement in a decent and calm setting. The study was performed after obtaining a license from ethic committee of Shahid Beheshti Medical Science University.

## 3. Findings

The participants were 18 women with perinatal loss experience who were interviewed after two weeks - three years after the sad event. Age range was 19 to 42 (mean 30.47 ± 6.35). Number of pregnancies ranged from 1 to 4 and gestational age was 16 to 40 weeks (26.92 ± 8.6). In four cases, the pregnancy terminated for medical reasons. The education level of the participants was as follows, middle school 5 people, High school graduate 5, and College graduate 8. 12 women were housewives, 5 women employees and one woman Self-employed.

2 mother had experienced abortion, 6 had experienced a stillbirth, and 10 had experienced a neonatal death Based on data analyses, 46 codes, 10 category, 18 subcategory, and 5 themes were extracted (Table 2).

**Table 2:** Category, subcategory, theme, and part of participants’ statements

Theme	Category	Subcategory	Participants’ Statements
**Beyond Pain**	**Pain and suffer**	Pain and disability	My breasts were hugely swollen and painful, I was not able to move… every move was a great pain, they were five or six times larger. (40 years old, G3 woman, one premature neonatal death and one spontaneous abortion)
	**A sense of loss**	Milk leakage as a annoying reminder of the loss	I managed to forget the loss until milk leakage started. It made me considerably sad and I was not able stop crying. (29 years old woman, G3 one IUFD at 32 week and one spontaneous abortion)
		Pain beyond physical pain	It was not only the physical pain that bothered me, feeling pain in my breasts and knowing that there was milk and having no child to feed was agonizing. (24 years old, G2, one premature neonatal death and one spontaneous abortion)
**longing for being a mother**	**Sense of motherhood**	Expecting a child to hug	Any mother loves having her child beside her, … there was milk but no child, It was very sad. (28 years old, G3, one premature neonatal death and one spontaneous abortion)
		Dreaming	I had a dream, in it they put my child before me told me give milk to him, then I woke up, my breasts were swollen and then came milk (perinatal loss, 32^nd^ week, 30 years old, 3 month after the event)
	**Motherhood Identity**	milk production capability	One of my friends told me not to press the breast because if I do, it leaks milk… I did opposite and milk came out and spread on my cloth. It was terrible, I kept pressing and cleaning the milk with a handkerchief (24 years old, two premature neonatal death)
		Controversial thoughts	Well… it was Ok at first [when the milk leaked], but then it was very sad because there was no infant to feed. (19-year old– one premature neonatal death)
**Coping Strategies**	**Medicine and non-medicine strategies**	medical intervention to sooth the pain	They prescribed medicine (34-year-old, G1, one IUFE at 25 week gestation death)
		nonmedical intervention	I used electric milk pump and massaging (34-year-old, one premature neonatal death)
	**Complete and comprehensive support**	Family members support	Well, my husband was helping me and it was very valuable, he was a very good companion. (34-year-old, one premature neonatal death)
			My breasts were hard and painful, that was terrible… my mother was helping me by pressing my breast to cut the pain (23 years old, neonatal death seven days after birth)
		Others’ support and comment	She told me to take care of it and try to eat less cold-natured foods?) 24-year-old-G2, abortion at 16 week gestation)
			My breasts were swollen and my sister in law compressed it with ice. (24-year-old - intrauterine death at 40 weeks of gestation)
**Expecting To Be Informed**	**Lack of knowledge about milk leakage and remedies**	Shocked	Then I asked by sister in law, what is this? What should I do? (24-year-old - G2, abortion at 16 week gestation)
		Puzzled	Someone told me to pump out the milk, but I was not able to touch it, it was the most horrible experience(40 years old, G3 woman, one premature neonatal death and one spontaneous abortion)
	**Need to know how to kill the pain and stop milk leakage**	Stopping milk leakage using medicine	They gave me medicine to stop the milk, but it still leaks and it is painful when I a laugh(29 years old woman, G1 one IUFD at 25 week and one spontaneous abortion)
		Expecting help and medicine to stop milk leakage	They did not give me anything for the leakage and I did not do anything. I was not able to clasp my arm, milk was collected in my armpit(29 years old woman, G3 one IUFD at 32 week and one spontaneous abortion)
**Beliefs And Values**	**Beliefs**	The infant food is already available	Relatives who came to visit said that the infants was a blessed one. (34 years old woman, G3, one premature neonatal death and one IUFD)
		Milks remains in breast for the next baby	My neighbor told me do not press your breasts and keep the milk and other vitamins for the next baby (24 years old, two premature neonatal death)
	**Values**	Why the milk leads	I kept thinking and telling God why his food is here already when he is not here. (34 years old woman, G3, one premature neonatal death and one IUFD)
		Religious approach to sooth the pain	My husband kept telling me… put yourself in Robab’s (Note 1) shoes, think about her when she lost Ali Asghar (Note 2) … how hard would have been for her when her child was not there for breastfeed, (34-year-old, one premature neonatal death).

### 3.1 Beyond Pain

Majority of the participants had experienced different levels of pain and sensitivity of the breasts between 2^nd^ and 5^th^ day after perinatal loss. Some also reported fever and the majority mentioned milk leakage for one to two weeks. A minority even reported long-term leakage (40 days or more). Mothers who had breastfeeding experience reported more milk leakage and breast engorgement, but the women with prenatal loss and without the experience of breastfeeding, did not expected the leakage and, thus, they were shocked and confused. One of the participants said:

“I was taking shower when I noticed something is coming out of my breasts. I called my sister in law and told her that my breasts were hard and painful and something white was coming out of them. Suddenly she burst into tear and said that it was milk.” (24-year-old - G2, abortion at 16 week gestation).

The participants had found milk leakage in different situations (e.g. under shower, in graveyard, while talking with others, and the like). However, for a great majority, the first leakage took place in their sleep and they had noticed the leakage after waking up. For instance, one of the participants said:

“Then my husband and I went to Behesht Zahra (graveyard)… he told me to stay in the car so I moved to back seat to have nap and I saw milk when I woke up.” (19-year old – one premature neonatal death)

Many of the participants who had breastfeeding experience mentioned that their milk was much more than their previous delivery so that while they did not have enough milk to breastfeed their previous child, they had too much milk and there was no child to feed. They expected an answer for this and somehow complained God for this.

For instance, one participant said:

“It was nothing like this when I had my second child and I barely had enough milk to feed him…, thanks God, I did not have milk for that one, and now my breasts are full of milk.” (28-year old–one premature neonatal death).

### 3.2 Painful Reminders

Milk leakage for majority of the participants was a painful reminder of the loss. Seeing milk and absence of the baby intensifies the sense of loss. Some of the comments in this regard are listed in Table 2. Other environmental factors such as hearing/seeing other infants triggered milk leakage in some cases. Milk leakage while having visitors at home was another painful reminder. According to the participants, visitors, in some cases, made them wonder if their child was dead or in other cases increased the mother’s tendency to isolation.

One case mentioned:

“Everyone expressed their sympathy and sorrow and that this was no one’s wished. Their comments were painful and hearing them increased milk in my breasts. After seeing a crying baby that they had with them, my breasts started to leak…they kept telling me that the baby is not alive. I told them that I have seen him and they responded if I was sure? They said if your breasts have milk, then the baby is alive; then I wondered, did I bury my child alive.” (29 years old woman, G3 one IUFD at 32 week and one spontaneous abortion)

**Longing being a mother**

Majority of the participants mentioned that they longed having their child beside them when they saw milk leakage and that other mothers were enjoying breastfeeding their own child. Absence of their child created considerable sense of envy and sorrow. (Table 2)

**Recognition as mother**

Majority of the participants who had been pregnant for more than 16 weeks reported milk leakage without stimulation. Some cases, however, had intentionally stimulated their breasts to prove their lactation capability. Some argued that doing this was helpful and calming.

**Breastfeeding dream**

Some of the participants reported that they had dreams about breastfeeding their infants and felt great sorrow when they woke up and saw their child was not there.

**Coping Strategies to sooth pain and milk leakage**

The participants had adopted different approaches to breast engorgement pain. Methods to deal with milk leakage and breast pain included sucking the milk, taking warm shower, ice compress, breastfeeding other children, breast bandage, using electric or manual milk pump, warm and cold compress, and asking help from experienced members of the family. Some participants had followed physician’s prescription and medication. Few cases had following avoiding strategies; one participant said:

“My breasts were bothering me for the first month afterward. I was reluctant to attend events where probability of encountering an infant was high. My breasts would leak milk when I was watching an infant, which was really agonizing.” (29 years old woman, G3 one IUFD at 32 week and one spontaneous abortion)

Complete support by the family member was one of the approaches followed by family members to sooth the pain of breast engorgement and pain. Recommending cold food, helping with pressing breast, ice compress, expressing sympathy and support and relying on religious beliefs were some of the measures. (Table 2)

**Expecting to be informed**

Some of the participants had been provided with enough information by the physician so that they expected the problem. However, in most of the cases, the mothers were not aware of the situation or the recommended remedies (e.g. bandaging breasts or medicines) were not effective. All cases reported milk leakage and many participants emphasized on necessity of more information.

Majority of participants did not seek help from health care provider when encountered with the breast problems and milk leakage. Among the reasons for this, lack of trust in the staff, no access to the physicians during holidays, tiredness, and simultaneousness with other problems are notable. Therefore, the participants tended to use home remedies to solve their problem. One of the participants mentioned:

“I thought, the doctor wouldn’t care even if I asked.” (24 years old, two premature neonatal death)

**Beliefs and values**

There are variety of beliefs and values regarding breast engorgement and milk leakage among mothers. Beliefs regarding the effects of milk leakage on future pregnancy, that milk leaks only when the child is alive, the milk is right of the lost infant, preventing milk leakage to save vitamins and minerals for future pregnancy, using cold foods to reduce milk leakage are some to notice. Religious beliefs such as God’s will and finding similarities with renowned religious stories and characters (e.g. Imam Hossein and his mother) were of other approaches that some participants followed to deal with hardship and sorrow of milk leakage after perinatal loss. (Table 2)

## 4. Discussion

Lactogenesis is an unrecognized aspect of what is experienced by the mothers who experience perinatal loss. (Moore, 2009)

The findings showed that perinatal loss after 16^th^ week of pregnancy results in milk leakage and breast engorgement between 2^nd^ and 5^th^ day after pregnancy. This is consistent with Pollard and Wambach (2012), Riordan and McGuinness et al. (2014).

### 4.1 Beyond Pain

The results showed that many mothers in the study suffered from breast engorgement and milk leakage. The problems that these mothers encountered with were beyond the pain of breast engorgement and leakage; so that every drop of the leaked milk was a reminder of the loss. This is consistent with McGuinness (McGuinness et al., 2014), and Chen et al. (2015).

Consistent with McGuinness, the participants noted increase of milk leakage during sleep. Increase of prolactin in blood stream in sleep (Donaldson-Myles, 2003; McGuinness et al., 2014) might be a probable cause of milk leakage.

Some of the participants mentioned hearing an infant’s crying or others’ chats about the loss as cause of increase of milk leakage, which might have been due to increase of stress and sorrow. Lawrence (2011) posited that mental factors and stressors might increase prolactin level. Other studies have reported different levels of the brain activity when the mother hears her infant’s crying during the first few month after delivery (Kim et al., 2011). However, there is no report of brain activity change in women after perinatal loss in response to hearing other infants crying. This is probably due to mental engagement and dreaming about breastfeeding their lost infant. This is a fruitful area of further research. There is also no report by other studies of milk leakage when mothers with perinatal loss see other small babies or talk about the event. The findings here may refer to cultural differences of the study population, which calls for visiting the mothers after she is discharged from the hospital by relatives and friends. Scientific findings also indicate that several factors such as biological, mental, cultural, and social factors have to with mother’s lactation problems during the first days and weeks after delivery.

### 4.2 Longing for Motherhood

For many women, being mother is a source of identity, pride, and success (Vallido et al., 2010). Losing an infant is more than mere loss and it is attached with many other failures such as losing the chance of being a mother (Leon, 1992). Therefore, pain and tenderness of breast and milk dripping are reminder of the potential mother for the woman who has lost her infant. These changes remind the mother that she could have been a mother and that there is no infant to feed, which brings more sorrow and envy.

### 4.3 Recognition as Mother

Some participants mentioned that seeing milk leakage was a pleasant and soothing feeling and even some had stimulated their breasts to ensure that they could produce milk. Groer wrote that breastfeeding is a pleasant experience for the mother and preserve the woman from many stressors. Kendall and Tackeet argued that lactation potentially is a defense mechanism against mood disorders after delivery (Kendall & Tackeet, 2007). However, soothing effects of lactation must be ascertained with further studies. Studies have shown that mothers construe milk leakage as recognition of the absent infant (Cole, 2012). Authors have reported that women who Women who had suffered perinatal loss tried to make others recognize her as a mother. However, there is no report of women’s reluctance to stop milk leakage as a way to have better understanding and feel the pain of perinatal loss. The inconsistent results can be explained by cultural differences. Findings by Welborn (2012) showed that denoting milk soothed pain of breast engorgement and milk leakage after perinatal loss (Welborn, 2012). However, none of the participants was interested in donating their milk, which could have been due to their concerns about religious beliefs (*Mahramiat)* and/or absence of milk bank in Iran (Nour-Mohammadi, 2015).

### 4.4 Dreams of Breastfeeding

Some of the participant had frequent dreams about feeding their infant and watching their baby grow. Studies have reported that dreaming is common among women after perinatal loss; however, other factors such as stress (Levin, & Nielsen, 2007) and stress after traumatic event (Kroth et al., 2004) are also mentioned as the reasons for such dreams.

### 4.5 Inadequate Information Provided by Health Personnel

Majority of the participants were not informed about probability of breasts problems, milk leakage and preventive measure, especially in late abortion cases. This is consistent with McGuinness (McGuinness et al., 2014). Therefore, there is a need to develop a general instruction to prevent milk leakage after perinatal loss. Spitz wrote that over the last century, small progress has been made in controlling milk leakage and breast problems. Moore argued that developed guidelines for perinatal loss are featured with a gap regarding milk leakage and breast problems. Our results highlighted the mothers’ need for emotional and training support by the health care provider to deal with emotional and physical problems of lactation.

In this studies few cases who educated about the problem. Majority of the participants did not seek help from health care providers to get rid of these problems after discharge from hospital. This is consistent with Chen’s study in Taiwan. The reasons that Iranian women do not seeking help from caregiver are tiredness, physical problems, and lack of access to physician; however, the participants in Chen’s study had stronger belief in traditional remedies; this indicates cultural differences (Chen et al., 2015).

The results indicated that majority of participants were not provided with enough educations as to controlling milk leakage. Failure to predict the breast engorgement or necessity of educations resulted in sustaining severe breast pain and confusion by women. Therefore and following Cole, there is a need to provide enough guideline to avoid problems of lactogenesis before discharging the women. Continuation of lactation causes considerable pain and pressure on breasts. (Sa’flund et al., 2004)

The results indicated that a small number of health personnel had recommended bandaging the breasts to avoid milk leakage and in rare cases, medical interventions had been prescribed. However, despite all these interventions, most of the participants reported breast engorgement and milk leakage. Other studies have confirmed that bandaging breast is not very effective and even some have reported that bandaging breasts increases chance of mastitis (Fetherston, 1998; Stift & Janke, 2003; Cole, 2012). To facilitate recovery process, there is a need to control physical and mental problems of women who experience perinatal loss; however, a specific protocol to prevent and solve breasts engorgement and milk leakage is absent. Therefore, physicians rely on their experience to help their patients.

### 4.6 Coping Strategies to Reduce Breasts Pain and Milk Leakage

The participants had adopted variety of solution to reduce the pain of breasts engorgement. Pressing breasts, milk pump, hot shower and bandaging breasts, ice compress, and medicine were some of these solutions. Other studies have reported that non-medicine controlling methods are also common such as reducing breast stimulation (Lawrense & Lwerense, 2014), using tight corset for 6hrs after delivery, avoiding hot weather, and ice compress (Cole, 2012; Lawrense & Lawrense, 2014); Chinese food diet (Pugmire, 1999); reducing liquid intakes, and some traditional medicine (Chen et al, 2015). Only one case mentioned using frozen lettuce on her breasts and without mentioning specific food, most of the participants noted that they have been advised to eat more cold-natured foods to stop lactation.

The results also indicated and family members and friends have considerable role in soothing the pain and many participants mentioned that they had asked help from their relatives and friends. The role of friends and relatives has been less emphasized by other studies, which indicates cultural differences. For instance, Chen showed that the participants were reluctant to consult with others about their problems in this regard (Chen et al., 2015).

Many participants noted that their first encounter with milk leakage was in bathroom and under shower, which is probably due to contact with hot water. In addition, some participants mentioned that they had hot shower to sooth the pain or stop the milk leakage. Although this method has short-term effect to sooth the pain, studies recommend that hot water stimulate breasts (Busta-Moore & Catlin, 2003). Using hot shower, probably, was the reason that the milk leakage in our participants was longer than what is reported by other works. Therefore, health personnel should warn the women about taking hot shower.

### 4.7 Beliefs and Values

Beliefs and values had considerable role in reducing or intensifying tensions of life. Some religious beliefs were helpful in reducing pains and problems of milk leakage. The role of religion in dealing with many stressful situations has been confirmed by many studies (Ano, & Vasconcelles, 2005; Harris et al., 2008; Pargament et al., 2004). Many participants took the event as a divine test and the fate and remained grateful for their blessing. There is the belief that religion plays a key role in coping with many stressful situations including all different kinds of grief; including the perinatal grief (Anderson et al., 2005; Cowchock et al., 2010). Although, religious beliefs have positive effects on reducing pains and sorrows, wrong beliefs increase stresses and problems in some participants. For instance, the belief that milk is produced only when the child is alive induces doubts in the mother if her child is alive or not, which may result in isolation. Therefore, public informing by mass media, for instance, may reduce wrong beliefs about milk leakage, pains, and sorrows experienced by such women.

### 4.8 Advantages of the Study

Direct interview with mothers who experienced perinatal death after 16^th^ week of pregnancy is an advantage of the present study. Previous studies have been limited to participants with more than or equal to 20 weeks’ gestation. Another advantage is that the research was qualitative studies, which increases opportunity of sharing experiences.

The qualitative studies approach, which gives more opportunities to participants for expressing and sharing their experiences, is another advantage of this study.

### 4.9 Limitations of the Study

Small number of participant, retrospective nature of the study, and reluctance of some women to share experiences with other to avoid remembering painful experiences are some limitations of the studies. Therefore, generalization of the results must be done with cautious.

## 5. Conclusion

The breasts engorgement and milk leakage intensify physical and mental problems, tendency to isolation in women, and even lactation problem in future pregnancies, which in turn prepare the ground for depression. It is recommended, therefore, to inform and educate mothers who experience perinatal loss about probability of milk leakage and breasts engorgement. It appeared that the mothers who did not received the required educations were those who had been discharged from maternity ward after losing their child in NICU ward so that at the time of encountering with the problems, no midwives and obstetrician were available. Therefore, pediatrician must give enough information to this woman before discharging them or refer the patients to obstetrician for consultation.

There is a need to launch a campaign in the mass media to improve public awareness about milk leakage after perinatal loss and avoid negative comments by others. Religious and medical consultation about breastfeeding and using religious beliefs to sooth sorrow and pains after perinatal loss are recommended in clinics and health services centers.

Eventually, it is notable that the present qualitative study was mostly aimed at providing richer information about lactogenesis, how to deal with the pains, and milk leakage after perinatal loss. Obstacles on the way of helping and proving the patients with guideline and medical consultation regarding milk leakage control after perinatal loss can be subject of future studies.
